# 

**Published:** 1902-10-15

**Authors:** 


### CONTENTS.

Some Dental History, - - *By Dr. A. T. Metcalf* 487
Solidified Formaldehyde, *By F. B. Lawrence, D.D.S.* 498
The Use of Mat Golds - - - - - - 508
Cocain and Its Many Uses in Dentistry,
*By Elliott R. Carpenter, D.D.S.* 515
Can the Pulp be Anesthetized by Injecting Cocain into the Peridental Tissues?
*By T. W. Brophy, M.D., D.D.S.* 518
Extracting the First Permanent Molars,
*By Dr. H. A. Pullen* 522
Orthodontia, - *By E. H. Angle, M.D., D.D.S.* 528
Work done at Fort Presidio by the Dental Corps,
*By J. S. Marshall, D.D.S.* 530
Editorial, - - - - - - - - - 533
Obituary, - - - - - - - - - - 535
Bibliography, - - - - - - - - - 538
Indents, - - - - - - - - - - 540

